# Guilt, shame, and postpartum infant feeding outcomes: A systematic review

**DOI:** 10.1111/mcn.13141

**Published:** 2021-01-24

**Authors:** Leanne Jackson, Leonardo De Pascalis, Jo Harrold, Victoria Fallon

**Affiliations:** ^1^ Department of Psychological Sciences, Institute of Population Health University of Liverpool Liverpool UK

**Keywords:** breastfeeding, infant feeding, infant formula, maternal mental health, postpartum, systematic review

## Abstract

Negative maternal affect (e.g., depression and anxiety) has been associated with shorter breastfeeding duration and poorer breastfeeding intention, initiation, and exclusivity. Other affective states, including guilt and shame, have been linked with formula feeding practice, though existing literature has yet to be synthesised. A narrative synthesis of quantitative data and a framework synthesis of qualitative and quantitative data were conducted to explore guilt and/or shame in relation to infant feeding outcomes. Searches were conducted on the DISCOVER database between December 2017 and March 2018. The search strategy was rerun in February 2020, together yielding 467 studies. The study selection process identified 20 articles, published between 1997 and 2017. Quantitative results demonstrated formula feeders experienced guilt more commonly than breastfeeding mothers. Formula feeders experienced external guilt most commonly associated with healthcare professionals, whereas breastfeeding mothers experienced guilt most commonly associated with peers and family. No quantitative literature examined shame in relation to infant feeding outcomes, warranting future research. The framework synthesis generated four distinct themes which explored guilt and/or shame in relation to infant feeding outcomes: ‘underprepared and ineffectively supported’, ‘morality and perceived judgement’ (breastfeeding), ‘frustration with infant feeding care’ and ‘failures, fears and forbidden practice’ (formula feeding). Both guilt and shame were associated with self‐perception as a bad mother and poorer maternal mental health. Guilt and shame experiences were qualitatively different in terms of sources and outcomes, dependent on infant feeding method. Suggestions for tailored care to minimise guilt and shame, while supporting breastfeeding, are provided.

**Key messages**

- Guilt is more prevalent among formula feeding mothers than among breastfeeding mothers. Sources of guilt also differ by infant feeding method.
- Framework synthesis identified the following themes: ‘underprepared and ineffectively supported’, ‘morality and perceived judgement’ (breastfeeding), ‘frustration with infant feeding care’, and ‘failures, fears and forbidden practice’ (formula feeding).
- Analyses identified a need for realistic, nonjudgemental, mother‐centred support (breastfeeding) and a need to provide emotional and practical support about safe formula feeding practice (formula feeding).
- A shift is recommended from a ‘6 month exclusive breastfeeding’ to an ‘every feed counts’ approach to providing breastfeeding support.

## 1 INTRODUCTION

Breastfeeding provides health benefits to infants, such as reduced risk of infectious morbidity and mortality, dental malocclusions, and overweight and diabetes later in life (Victora et al., 2016). Breastfeeding also protects mothers from breast and ovarian cancer and reduces risk of diabetes (Victora et al., 2016). As such, the World Health Organisation (WHO) recommend exclusive breastfeeding for the first 6 months postpartum (UNICEF, 2017). Despite awareness of breastfeeding benefits and promotional campaigns (Thomas, 2014), WHO recommendation compliance remains poor in developed countries.

A systematic review involving 11 European countries completing a standardised national survey found that in all participating countries, breastfeeding rates declined gradually from initiation after birth to 1 to 2 months postpartum and at 6 months postpartum (e.g., in the Netherlands, from 80% initiation, to 64% prevalence at 2 months postpartum, and 51% prevalence at 6 months postpartum; Theurich et al., 2019). A similar decline can be seen between breastfeeding initiation and breastfeeding duration in other developed countries, including Australia (Australian Government: Department of Health, AGDH, 2019), Canada (Chalmers et al., 2009), United Kingdom (McAndrew et al., 2012) and the United States (Center for Disease Control and Prevention, CDCP, 2019). Given these trends, it is important to explore potential factors contributing to the gap between breastfeeding initiation and breastfeeding prevalence at 6 months postpartum in developed countries.

Maternal emotional state is a modifiable factor which affects breastfeeding outcomes. In a systematic review of 48 studies, higher postpartum depressive symptomatology was significantly associated with shorter breastfeeding duration and early exclusive breastfeeding cessation, compared with mothers reporting fewer depressive symptoms (Dias & Figueiredo, 2015; see also Dennis & McQueen, 2009). A narrative synthesis of 33 studies indicated higher postpartum anxiety was associated with reduced likelihood of exclusive breastfeeding and increased risk of early breastfeeding cessation, compared with mothers reporting fewer anxiety symptoms (Fallon, Groves, Halford, Bennett, & Harrold, 2016). High prenatal anxiety was also associated with reduced breastfeeding intention and exclusivity (Fallon, Bennett, & Harrold, 2016; Grigoriadis et al., 2018).

Guilt has also been an associated outcome of infant feeding, and especially so for formula supplementation. Guilt has been defined as feelings of remorse concerning a moral transgression (Niedenthal, Tangney, & Gavanski, 1994). In existing literature, formula feeding was perceived as a moral failing, as maternal discourse was frequently spoken of synonymously with having not done ‘right’ by one's infant (Brodribb, Fallon, Jackson, & Hegney, 2010; Lakshman & Ong, 2009) and with having failed to meet expectations of oneself postnatally (Kair, Flaherman, Newby, & Colaizy, 2015). Such feelings of guilt have been reportedly exacerbated by breastfeeding education and promotion which inefficiently prepares women for postnatal infant feeding difficulties (Groleau, Pizarro, Molino, Gray‐Donald, & Semenic, 2016). Guilt has also been associated with feelings of anger being held towards healthcare professionals, when mothers perceived that they had received ineffective support (Humphries & McDonald, 2012).

Perceiving that healthcare professionals were promoting breastfeeding as a moral obligation and perceiving that breastfeeding was overly medicalised were both linked with guilt and undermined maternal autonomy (Benoit, Goldberg, & Campbell‐Yeo, 2016). Indeed, perceiving that formula feeding was risky to infant health and perceiving that one had moral responsibility over infant feeding method were both associated with feelings of guilt for women who were supplementing with formula (Taylor & Wallace, 2017; Williams, Donaghue, & Kurz, 2012). Interestingly, women who perceived that supplementing with formula milk was not their decision did not experience guilt to the same degree, highlighting the importance of perceived responsibility in determining the presence or absence of maternal guilt (Holcomb, 2017).

Shame also occurs in association with infant feeding experiences. Shame has been defined as the internalisation of guilt to the self, especially if one perceives themselves to be failing in front of others (Niedenthal, Tangney, & Gavanski, 1994). Although both guilt and shame concern a perceived or actual moral transgression, guilt is externalised and behaviour‐orientated, whereas shame concerns the internalisation of said transgression to the self (Niedenthal, Tangney, & Gavanski, 1994). Taylor and Wallace (2012) further supported this definition in finding that globalised assessments of the self as a bad mother, in association with formula feeding practice or public breastfeeding, exceeded the behaviour‐focused feelings of guilt and instead focused on the self as a failing entity. In infant feeding literature, feeling that one was failing their moral obligation to breastfeed when challenges were experienced, and feeling like one was failing in front of others, were both linked with feelings of shame (Hanell, 2017). For breastfeeding mothers, objectification of infant feeding was also associated with shame and distress (Thomson, Ebisch‐Burton, & Flacking, 2015).

In quantitative infant feeding literature, guilt has been examined through binary response options ‘yes/no’ in response to direct questions about feeling guilty due to one's infant feeding method (Chezem, Montgomery, & Fortman, 1997; Fallon, Bennett, et al., 2016; Komninou, Fallon, Halford, & Harrold, 2016). To the author's knowledge, there are currently no quantitative studies examining shame in relation to infant feeding outcomes. In qualitative infant feeding literature, guilt and shame have been identified in thematic analysis (e.g., identified theme ‘relief and guilt’ in Fahlquist, 2016, and ‘shame’ examination in Hanell, 2017) and have occasionally been grouped in thematic analysis (e.g., identified theme, ‘stress, shame and guilt’ in Asiodu, Waters, Dailey, & Lyndon, 2017). Framework analyses have also been used to offer a holistic picture of how shame is experienced in an infant feeding context, which have considered both individual vulnerabilities, for example, idealised expectations of ‘good mothering’, and social factors, for example, fears concerning breastfeeding in public (Thomson, Ebisch‐Burton, & Flacking, 2015).

Current literature evidences the relationship between poorer breastfeeding outcomes and negative maternal affect, such as anxiety, depression, guilt and shame, in developed countries. Although there are existing reviews examining the relationship between infant feeding outcomes and maternal anxiety and depression, guilt and shame literature has yet to be synthesised in relation to infant feeding outcomes. Understanding this relationship may allow better identification of women vulnerable to experiencing these emotions and provide recommendations for tailored care. Given the identified decline in breastfeeding prevalence at 6 months postpartum compared with initiation rates in developed countries (AGDH, 2019; CDCP, 2019; Chalmers et al., 2009; McAndrew et al., 2012; Theurich et al., 2019), the current review will synthesise data from developed countries, only. This mixed‐methods systematic review aims to (a) examine the relationship between guilt and/or shame and different infant feeding outcomes and (b) examine how guilt and/or shame are experienced differentially depending on infant feeding method.

## 2 METHOD

The current review was preregistered on PROSPERO in November 2018 (https://www.crd.york.ac.uk/PROSPERO/#recordDetails). A protocol was developed based on a scoping literature search.

### 2.1 Ethical considerations

Ethical approval was not required for the current study as it used secondary data collection and analysis. Findings from this study will form part of LJ's PhD thesis.

### 2.2 Eligibility criteria

Studies were included if they explicitly explored guilt and/or shame as variables or if they reported them as key themes in an infant feeding context and if they were conducted in developed countries, as defined by the Statistical Annex (Country Classification, 2014). Given cultural variation in breastfeeding practices and maternal wellbeing between developed (Leahy‐Warren, Creedon, O'Mahony, & Mulcahy, 2017) and developing (Wanjohi et al., 2017) countries, it was deemed appropriate to only include studies from the former. This is also supported by the identified decline in breastfeeding prevalence at 6 months postpartum compared with initiation rates reported in developed countries (AGDH, 2019; CDCP, 2019; Chalmers et al., 2009; McAndrew et al., 2012; Theurich et al., 2019). See Table 1 for inclusion criteria for study selection.

**TABLE 1:** Inclusion criteria for study selection, mapped on to PEO criteria

Population	Exposure	Outcome
Maternal age over 18 Infant born full term (>37 weeks gestation) Infants born of a healthy weight (>2,500 g) Singleton infants, only Maternal absence of clinically diagnosed mental distress e.g., postnatal depression, postnatal anxiety, postnatal psychosis, prenatal anxiety, or prenatal depression, unless controlled for in analysis Absence of maternal condition(s) which would otherwise affect ability to breastfeed, such as breast reduction surgery; pituitary dysfunction; untreated tuberculosis; hepatitis B and C; active herpes lesions; human immunodeficiency virus (HIV); and substance abuse (Sheknows, 2007) No feeding, physical, or mental congenital irregularities in infant which would otherwise affect feeding ability, for example, tongue tie, lactose intolerance, cleft lip	Studies must have been conducted in a developed country, as defined by the Statistical Annex (Country Classification, 2014) Guilt and/or shame must be explicitly explored in the context of postnatal infant feeding experiences (i.e., formula and breastfeeding intention, initiation, duration, method at time of investigation, and qualitative experiences with these outcomes) Data collected in the first 6 months of life Guilt and/or shame must be explicitly explored in study results section, either in thematic analysis or as an outcome variable	Examination of breastfeeding and/or combination feeding, and/or formula feeding initiation, exclusivity, and duration. Qualitative experiences of infant feeding Primary data collection Written in any language Grey literature and dissertations/theses Cross‐sectional and longitudinal designs Qualitative and quantitative methodologies
	
	
	
	
	
	
	
	
	
	
	
	

### 2.3 Search strategy

A search strategy was developed in line with Population Exposure Outcomes criteria (PEO; University of London, 2020; see Table 2). PEO criteria were utilised to develop clear study aims and research questions, as recommended by O'Harhay and Donaldson (2020) and in line with other attempts to answer health‐related questions (Davies, 2011). PEO criteria were also utilised to map inclusion criteria for article selection at title, abstract and full text screening stages. Key terms utilised in the final search strategy were determined via a scoping literature search and the subsequent identification of relevant key words included in identified papers. All named authors agreed upon the final search strategy.

**TABLE 2:** Population exposure outcomes (PEO) for exploring guilt and shame in relation to infant feeding outcomes

Review question(s)	Population	Exposure	Outcome
(a) Examine the relationship between guilt, shame, and infant feeding outcomes. (b) Explore how guilt and shame are experienced by mother's, dependent on infant feeding method.	Women who have given birth in the past 6 months to a full‐term (>37 weeks), healthy infant (>2,500 g). Absence of maternal or infant congenital abnormalities which would otherwise affect ability to breastfeed. Women with no clinical diagnosis of mental distress, unless controlled for in study analysis. When reported, absence of traumatic experiences e.g. history of sexual abuse, or significant displacement, which may otherwise affect emotional or practical infant feeding experiences.	To be included in the current analysis, included studies needed to involve participants with infants under 6 months of age, who have previously or are currently experiencing postnatal guilt and/or shame. As such, included articles needed to explicitly examine maternal guilt and/or shame in relation to infant feeding outcomes.	Formula and breastfeeding intention, initiation, duration and method at time of investigation. Qualitative experiences related to outcome measures were also explored.

Keywords used to search for articles included ‘shame*’; ‘guilt*’; ‘stigma*’; ‘moral*’; ‘breastfeed*’; ‘breast feed*’; ‘breast‐feed*’; ‘bottle feed*’; ‘bottle‐feed*’; ‘infant feed*’; ‘infant‐feed*’; ‘formula feed*’; ‘formula‐feed*’; ‘combi* feed*’ and ‘human lactat*’. Boolean operators were used to blend keywords, and truncation was used to identify variations of keywords. Articles were screened for suitability against eligibility criteria, outlined in Table 1, at title, abstract and full text stages. For an example of the search strategy being utilised in a single database, please see Appendix A.

Searches were conducted between December 2017 and March 2018. Interrater reliability was assessed by a second researcher who independently screened 25% of included articles generating an almost perfect unweighted kappa statistic of 0.933 (McHugh, 2012). Reference lists of included articles were systematically screened identifying three additional articles. Authors of included articles were contacted for inclusion of unpublished work(s) which identified 1 additional article. No date or language limitations were placed on the search strategy. The search strategy identified 7 papers which were written in French, 1 study which was written in Polish, 1 study which was written in Spanish and 1 study which was written in Portuguese. Studies not written in English were translated by independent researchers and screened using the outlined search strategy and inclusion criteria. The search strategy was rerun in February 2020 identifying 1 additional article.

**FIGURE 1:**
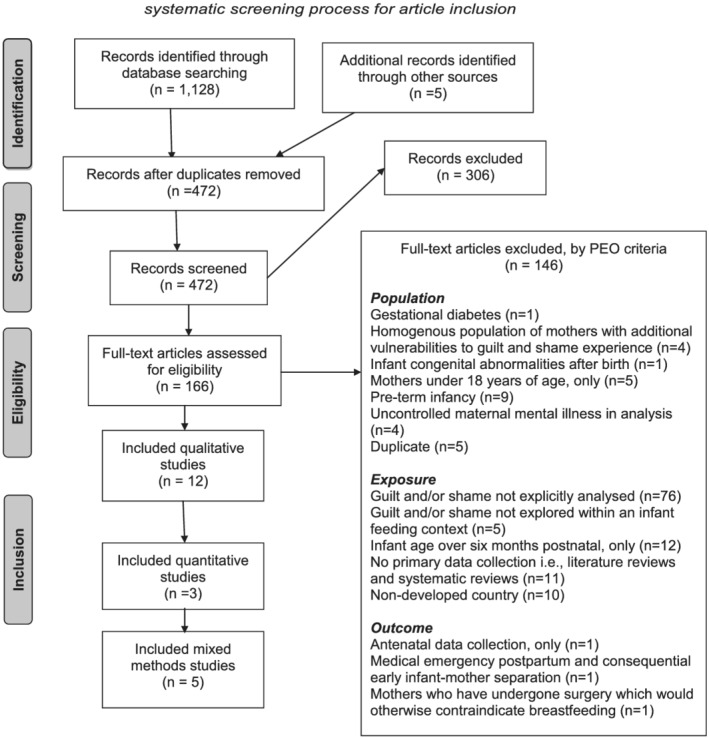
PRISMA 2009 flow diagram identifying three stage systematic screening process for article inclusion

During screening, 1 paper was identified which examined a sample of mothers who experienced breastfeeding aversion. It was decided to remove this paper due to associated feelings of shame which may have otherwise confounded findings (Morns, Steel, Burns, & McIntyre, 2020). An additional 2 papers involved samples of women who had a history of sexual abuse. These papers were excluded due to evidence suggesting that historic sexual abuse may affect parenting style and anxieties and may contraindicate breastfeeding comfort due to feelings of shame (Haiyasoso, 2019; Wood & Esterik, 2010). A further paper involved mother‐infant dyads who had been separated shortly after birth due to medical emergency. This paper was excluded due to subsequent interruption of breastfeeding initiation in the first hour of giving birth (Phillips, 2013). Finally, 1 study involved a sample of refugee women. This study was excluded due to evidence suggesting that this particularly vulnerable group have exceptionally inadequate access to social and healthcare professional support which may have otherwise confounded findings (Lerseth, 2013; Madanat, Farrell, Merrill, & Cox, 2007). See Figure 1 for Preferred Reporting Items for Systematic Reviews and Meta‐Analyses (PRISMA; Moher et al., 2009) diagram.

### 2.4 Quality assessment

The Standard Quality Assessment Criteria for Evaluating Primary Research Papers from a Variety of Fields (SQAC; Kmet, Cook, & Lee, 2004) was used for quality assessment. The SQAC contains separate point‐based checklists for quantitative and qualitative methodologies. Mixed methods studies were assessed using both checklists. Quality assessment was conducted by 2 researchers independently. Any discrepancies were discussed, and if agreement could not be reached, then a third member of the research team was consulted. Quality assessment framed suggestions made for future research.

### 2.5 Data extraction

Data extraction from the 20 included studies comprised references, aims and/or hypotheses, inclusion and exclusion criteria, sampling method and characteristics, drop‐out rate, design, location, methodology, outcome variables, descriptive statistics, analysis method, summary of guilt and/or shame findings, outline of guilt and/or shame definition, secondary findings, related keywords and methodological comments. The following information was extracted from quantitative studies only: control for confounders and exposure/outcome variable(s). Data extraction was conducted by 2 researchers independently. Any discrepancies were discussed, and if agreement could not be reached, then a third member of the research team was consulted.

### 2.6 Analysis

A narrative synthesis (Rodgers et al., 2009) of quantitative papers was conducted, due to the small number and heterogeneity of identified papers, to address research question. (a) Qualitative and quantitative studies were examined using framework synthesis (Ritchie, Lewis, Nicholls, & Ormston, 2003), to address research question. (b) Framework syntheses have been utilised in previous infant feeding literature (Thomson, Ebisch‐Burton, & Flacking, 2015). Stages of conducting a framework synthesis included familiarisation with methodology and results sections of included articles, construction of initial thematic framework, utilisation of framework to index and sort identified themes to address research questions and reviewing and refining applied framework for coherence. Because infant feeding practices were clearly reported in all included literature, data were synthesised in relation to infant feeding method (i.e., breastfeeding and formula feeding mothers) to enable the comparison of guilt and shame experiences. For mixed methods papers, quantitative components were included in the narrative synthesis, and qualitative components and relevant quantitative components were included in the framework synthesis. Data sharing was not applicable to this article, as no new data were created or analysed in this study.

## 3 RESULTS

After removal of duplicates, the search strategy yielded 467 studies dating 1948–2017, across 34 databases (see Table 3 for tabulation of article frequencies by database, before and after removal of duplicates). The study selection process identified 20 articles, published between 1997 and 2017. Of included literature, 13 studies examined guilt, 3 studies examined shame and 4 studies examined both guilt and shame. No included quantitative literature analysed shame. Included studies came from the following developed countries: UK (11 studies); USA (3 studies), online, open internationally (3 studies); Norway (1 study); France (1 study) and Sweden (1 study).

**TABLE 3:** Frequency table to display articles identified from search strategy per database, before (and after) removal of duplicates

Database	Number of identified articles before (and after) duplicate removal
Academic Search Complete	176 (142)
Agricola	6 (2)
America: History and Life with Full Text	5 (1)
Art & Architecture Complete	3 (0)
BioOne Complete	1 (1)
Books at JSTOR	3 (2)
British library EThOS	13 (9)
CINAHL Plus	126 (10)
Clinicaltrials.gov	11 (11)
Communication and Mass Media Complete	1 (0)
Complementary Index	199 (51)
Computers and Applied Sciences Complete	2 (0)
Dentistry and Oral Sciences Source	1 (0)
Digital Access to Scholarship at Harvard (DASH)	2 (0)
Directory of Open Access Journals	43 (15)
Education Research Complete	10 (0)
Emerald Insight	1 (1)
Environment Complete	13 (1)
ERIC	5 (1)
Global Health Archive	2 (2)
Historical Abstracts with Full Text	7 (4)
Humanities International Complete	12 (2)
Informit Health Collection	1 (1)
JSTOR Journals	12 (10)
KoreaScience	1 (1)
LexisNexis Academic: Law Reviews	3 (3)
Library, Information Science & Technology Abstracts	1 (0)
MEDLINE with Full Text	208 (43)
National Criminal Justice Reference Service Abstracts	1 (1)
Newswires	14 (6)
Oxford Scholarship Online	4 (4)
Persée	1 (1)
Philosopher's Index	6 (0)
Project MUSE	1 (1)
PSYCINFO	106 (29)
Research Starters	1 (1)
ScienceDirect	41 (40)
SciELO	12 (9)
SPORTDiscus with Full Text	7 (3)
SSOAR – Social Science Open Access Repository	7 (6)
Supplemental Index	40 (40)
SwePub	18 (13)
Teacher Reference Center	1 (0)

Of included literature, 12 studies used qualitative methodologies. Data collection methods were as follows: 5 studies used semistructured interviews, 3 studies used semistructured interviews and focus groups, 1 study used semistructured interviews with field observations, 1 study used a case study, 1 study used an auto‐ethnographical approach and 1 study used an online survey with open text responses. Of included qualitative literature, 3 studies used a longitudinal design, and 9 studies used across‐sectional design. Qualitative sample sizes ranged from (with *n* indicating the total number of participants involved in this form of data collection) 9 to 36 for semistructured interviews (*n* = 111), 51 to 78 for focus groups (*n* = 192), 2 studies used single unit participation (*n* = 2) and 1 study consisted of 2 qualitative online surveys, with 5 and 42 participants. Total qualitative sample size was 388. Given that only 2 included papers examined shame in relation to infant feeding outcomes, and neither of these included papers examined shame quantitatively, guilt and shame were grouped together in the framework synthesis, and results were split by infant feeding method.

Of the 3 included quantitative papers, 2 studies used a cross‐sectional, online methodology, and 1 study used a longitudinal, telephone questionnaire. Quantitative sample size ranged from 53 to 679. Total quantitative sample size was 1,333. The search strategy identified 5 mixed method studies, 4 of which used the same dataset (Lee, 2007a, 2007b, 2007c; Lee & Furedi, 2005) using a structured questionnaire and semistructured interviews. The fifth study involved quantitative analysis of telephone questionnaires and semistructured interviews. Sample size for mixed methods papers ranged from 12 to 33 for qualitative components (*n* = 45) and 86 to 504 for quantitative components (*n* = 590). Total sample size for mixed methods studies was 635. See Table 4 for summary table of included literature.

**TABLE 4:** Summary table to demonstrate extracted information of included articles in narrative synthesis of quantitative studies and framework synthesis of qualitative and quantitative studies

Study ID (reference)	Study aim(s)	Design	Sample information, location and attrition	Analysis	Findings	Methodological comments
Asiodu, I. V., Waters, C. M., Dailey, D. E., & Lyndon, A. (2017). Infant feeding decision‐making and the influences of social support persons among first‐time African American mothers. *Maternal and Child Health Journal, 21*, 863–872. https://doi.org/10.1007/s10995‐016‐2167‐x	Describe the infant feeding experiences of African American mothers and their support persons	*Qualitative* Semi‐structured interviews, field observations, longitudinal (antenatal interviews with postnatal follow up)	San Francisco Bay, USA 14 pregnant women and 8 support persons, no reported attrition	Braun and Clarke's (2006) theoretical and latent approach, thematic analysis; situational maps. Theoretical perspectives of Black Feminist Theory (Collins, 2008) and Family Life Course Development Theory (Bengston & Allen, 1993) informed the study. Longitudinal critical ethnographic approach taken to analysis	*Descriptive statistics* Opportunity sampling used to recruit participants. Participants were aged 21–36, half of the participants were married or partnered and employed, most were high school graduates *Qualitative themes* Best for baby; normalisation and role models; social support; fluid social dynamics and resiliency; seeking support and empowerment; combination feeding; and stress, shame, and guilt	Socioeconomically diverse sample of women recruited. Limited inclusion criteria which did not consider potentially confounding factors affecting breastfeeding ability and/or emotional experiences. Six participants did not have support persons, potentially biasing findings. Guilt and shame grouped in thematic analysis; no definitions provided to outline use of terms
Chezem, J., Montgomery, P., & Fortman, T. (1997). Maternal feelings after cessation of breastfeeding: Influence of factors related to employment and duration. *Journal of Perinatal and Neonatal Nursing*, *11*(2), 61–70	Measure feelings following weaning in women planning on returning to employment within the first postpartum year	*Quantitative* telephone interviews, longitudinal (third trimester antenatal, with postnatal follow ups at 6 weeks, 3, and 6 months)	Indiana, USA 53 participants, of 68 women approached (22.06% attrition). 6 did not breastfeed, and 9 could not be reached for all interviews and so were excluded	Pearson's correlation coefficients determined relationships between demographics and dependent variables, t‐tests determined differences in dependent variables and breastfeeding cessation motivation	*Descriptive statistics* Opportunity sampling used to recruit participants. Participants' age brackets ranged from below 20 years (3 participants) to 36–40 years (2 participants), 51% White, 94% primiparous *Findings* Women who did not meet infant feeding plans upon return to work scored significantly higher guilt (*p* = 0.004) compared with women who did met these goals	Small sample size which did not control for potential confounders in statistical analyses. T test statistics, degrees of freedom and effect sizes not reported. Unrepresentative sample of women (mostly 26–30 years old, White, and with a Bachelor's degree) recruited. Limited inclusion criteria which did not consider potentially confounding factors affecting breastfeeding ability and/or emotional experiences. Opportunity for expansion given for response option ‘other’ regarding reason for breastfeeding cessation, was not given for emotional response post‐cessation. Guilt measured using a 5‐point Likert scale (1 very guilty – 5 not guilty), no definition provided for guilt
Crossley, M. L. (2009). Breastfeeding as a moral imperative: An authoethnographic study. *Feminism & Psychology, 19*(1), 71–87. https://doi.org/10.1177/0959353508098620	Illustrate how breastfeeding can be fraught with tension as contradictory pressures pull women in different directions	*Qualitative* Dialogical interview between author and author's partner, case study, cross‐sectional	UK One participant, attrition not applicable	Lee and Fuerdi's (2005) paper informed conversation topics. Autoethnographic and feminist‐inspired (e.g. Stanley, 1993) social scientific approach taken (Richardson, 2000), thematic analysis. McAdams' (1993) methodological approach to narrative Psychology used to develop semi‐structured interview schedule	*Descriptive statistics* Autoethnographic approach. No descriptive statistics reported *Qualitative themes* Intention/motivation to breastfeed; things go wrong: Conflicting interpretations; bottle feeding, confusion, guilt, and shame; conflicting identities – The ‘relational’ mother vs. the ‘slim, fit person’	No evidence of inter‐rater reliability testing during analysis. Method used to conduct thematic analysis not reported. No reporting of demographic information. Guilt and shame grouped together in thematic analysis; no definitions provided for guilt or shame
Dalzell, J. (2007). *Exploring the infant feeding experiences of low‐income mothers and the support offered by health professionals involved in their care: A qualitative study* [unpublished Master's dissertation]. University of Dundee.	Explore and gain an understanding of women's breastfeeding experiences, and to explore the support offered by health professionals	*Qualitative* Semi‐structured interviews, Cross‐sectional	University of Dundee, UK 18 postnatal women and 10 health professions Of 23 mothers approached, 18 consented to participate	Interpretative (Bryman, 2001; Robson, 2002), phenomenological and positivist approach (Cormack, 2000), thematic analysis; ontological perspective (Porter, 1993; Crotty, 1998) framework analysis applied	*Descriptive statistics* Opportunity sampling used to recruit participants. No descriptive statistics reported *Qualitative themes*initiating breastfeeding; supplementary feeding; separation of mothers and babies; developing and maintaining milk supply; support at home; being able to breastfeed in a community; feelings	Good consideration of theoretical underpinnings for analysis approach. Deprivation index used to recruit mothers from disadvantaged areas so to meet study objectives. Clear exclusion criteria included so to avoid potential confounders. Greater number of breastfeeding mothers recruited than was intended due to participant misclassification of infant feeding method. ‘Feelings’ is a very broad theme which could have been broken up further. No definition of guilt provided
Fahlquist, J. N. (2016). Experience of non‐breastfeeding mothers: Norms and ethically responsible risk communication. *Nursing Ethics, 23*(2), 231–241. https://doi.org/10.1177/0969733014561913	Understand how non‐breastfeeding mothers experience breastfeeding and breastfeeding discontinuation	*Qualitative* Online study with open text responses	Sweden, UK, and the Netherlands Two online surveys. Survey 1 had 5 respondents. Survey 2 had 42 respondents No attrition information reported	Content analysis	*Survey 1* Purposeful sampling used to recruit participants. 4 Swedish women and 1 English woman participated in survey one. *Survey 2* Purposive sampling used to recruit participants. 24 women gave birth in Sweden, 7 in the UK, and 4 in the Netherlands. 1 woman did not provide details about country gave birth in. All but 4 respondents tried to breastfeed initially. No demographic information reported *Qualitative themes* Depression, anxiety, and pain – Feeling like a failed mother; relief and guilt; bonding problems; and feeling trapped – Losing one's freedom	No specific information regarding how included items were selected for in the distributed surveys. No information regarding approach taken to conducting the content analysis reported. No reporting of demographic information. Some themes are lesser supported than others e.g. ‘losing one's freedom’ is much lesser supported than ‘relief and guilt’. The theme ‘depression, anxiety, and pain – Feeling like a failed mother’ has many sub‐components which could have been examined independently. One participant spoke of feeling, ‘ashamed’ (p. 236), however, this account is under the ‘relief and guilt’ theme. No definition of guilt provided
Fallon, V., Komninou, S., Bennett, K. M., Halford, J. C. G. & Harrold, J. A. (2016b). The emotional and practical experiences of formula‐feeding mothers. *Maternal and Child Nutrition, 13*(4), 1–14. https://doi.org/10.1111/mcn.12392	Describe the emotional and practical experiences of formula feeding mothers	*Quantitative* online, cross‐sectional	Online 601 mothers of infants up to 26 weeks of age, of 890 potential participants. 289 were excluded for survey non‐completion (32.47% attrition)	Relative risk ratios conducted for association between exposure and outcome variables, using multinomial logit models. Backward elimination used to build adjusted models	*Descriptive statistics* Purposive sample recruited. Participants were aged 18–46, 64% were married, 62% primiparous, 57.2% from the UK (1.2% Irish, 20.3% USA, 9.5% Australian, 3.7% new Zealanders, 5% Canadian, 2% other, European, and 1.1% other, world). Demographic variables kept as confounders if they changed the beta coefficients of the exposure categories by more than 10% *Findings* Guilt experienced by 67% of mothers; relative risk of exclusively formula feeding was four times lower compared with women who intended to exclusively breastfeed but whom were exclusively formula feeding at the time of investigation (RRR: .45, 95% CI: .25, .79) and 2 times lower in combination feeders (RRR: .38, 95% CI: .21, .64) for those experiencing guilt	Addressed a comprehensive range of potential confounders. Binary examination of guilt as present or absent. Lack of survey item validity testing. Unrepresentative sample of mostly primiparous women who were wither married or living with a partner. Participants asked about guilt via binary ‘yes/no’ response options to question, ‘have you ever felt guilty about the way you choose to feed your baby?’ (p. 6) No definition of guilt provided
Fox, R., McMullen, S., & Newburn, M. (2015). UK women's experiences of breastfeeding and additional breastfeeding support: A qualitative study of baby café services. *Pregnancy and Childbirth, 15*(147), 1–12. Https:doi.org/10.1186/s12884‐015‐0581‐5	Identify which elements of baby café support are effective and why, and to examine how such these services can be integrated with other forms of support	*Qualitative* Interviews and focus groups, cross‐sectional	UK (London, south East England, northern England), 36 interviews and 5 focus groups with 51 mothers, 1 of 9 sites approached declined participation, 12 women approached refused participation	Inductive approach, thematic analysis	*Descriptive statistics* Convenience sample recruited. Participants were aged 23–44,33 primiparous and 18 multiparous, mostly highly educated and employed, all but one was cohabiting, 10 born outside of the UK *Qualitative themes* Antenatal education: Unrealistic expectations; realistic experiences: Pressure, guilt, and blame; postnatal care: Conflicting advice and undermining of confidence; support from friends and family; seeking breastfeeding support; breastfeeding role models; breastfeeding as a journey	Evaluation steering group consisting of support group facilitators oversaw the research being conducted, meeting regularly throughout the conduction of the study. Sites were selected to reflect diverse locations and support provision. Method of data analysis clearly outlined. No clear exclusion criteria for participant recruitment. Interviews were conducted in baby Cafés with group facilitators present which may have led to social desirability bias. Sample was biased towards older mothers, and those with higher levels of education and employment. No definition of guilt provided
Hanell, L. (2017). The failing body: Narratives of breastfeeding troubles and shame. *Linguistic Anthropology, 27*(2), 232–251. https://doi.org.liverpool.idm.oclc.org/10.1111/jola.12158	Explore the relationship between discourse and difficult breastfeeding experiences, and proposing that shame arises in deviations from bio‐political ideologies	*Qualitative* Case study, longitudinal (6 monthly interviews, and regular contact through email, text, and Instagram)	Stockholm One participant, attrition not applicable	Field notes made on text‐based communications and photographic reports included alongside longitudinal narrative analysis (Bauman, 2004; Foucault, 1991)	*Descriptive statistics* No information on sampling strategy provided. Participant was in her late 20s, primiparous, lived with husband, preschool teacher *Qualitative themes* Discourse and malfunctioning breastfeeding; online searches; medicalisation; embodiments of bio‐political regimes; failing before the other; inviting the medical gaze; creating the experience in narrative	No information reported regarding study design. No information reported on how interview schedules were developed, or on method of analysis conducted. No evidence of inter‐rater reliability being checked during analysis. No definition of guilt provided. Ahmed's (2014) and Bourdieu's (2001) definitions of shame utilised
Hvatum and Glavin (2017). Mothers' experience of not breastfeeding in a breastfeeding culture. *Journal of Clinical Nursing, 26*, 19–20. https://doi.org.liverpool.idm.oclc.org/10.1111/jocn.13663	Investigate the experience of not breastfeeding in a breastfeeding culture	*Qualitative* Descriptive design with semi‐structured interviews, cross‐sectional	Norway, 12 mother‐infant dyads, no reported attrition	Graneheim and Lundman's (2004) approach, thematic analysis	*Descriptive statistics* Opportunity sampling technique used to recruit participants. Participants were aged 25–40, all infants singleton and healthy, all bar one were married/cohabiting *Qualitative themes* Desire to adopt Norwegian culture; feeding as though one was breaking the law; and lack of and unbalanced information	Inclusion criterion, ‘mothers who had experienced stressful breastfeeding’ (p. 3146) is quite vague. No consideration for potential congenital abnormalities which may otherwise contraindicate breastfeeding. Two mothers provided retrospective accounts of their infant feeding experiences dating three years prior. Good outline of interview schedule provided. Method of analysis outlined clearly. No evidence of inter‐rater reliability testing reported. Guilt and shame grouped in thematic analysis. No definitions of guilt or shame provided in text. Guilt accounts and shame accounts are clearly distinguished in results section
Komninou, S., Fallon, V., Halford, J. C. G., & Harrold, J. A. (2016). Differences in the emotional and practical experiences of exclusively breastfeeding and combination feeding mothers. *Maternal and Child Nutrition, 13*, 1–11. https://doi.org/10.1111/mcn.12364	Quantify the emotional and practical experiences of breastfeeding mothers	*Quantitative* online, cross‐sectional	Online 679 mothers with infants up to 26 weeks of age, of 845 potential participants. 151 were excluded for survey non‐completion (19.64% attrition). 7 exclusively formula feeding mothers excluded due to statistical issues with small sample size.	Relative risk ratios for the association between exposure and outcome variables calculated using binary logit models. Backward elimination used to build adjusted models	*Descriptive statistics* Opportunity sampling used to recruit participants. Participants were aged 19–45, 95.8% were married or living with their partner, 88.1% from the UK (other countries of residence not reported), 45.8% primiparous, and 90% in paid occupation. Demographic variables were kept as confounders if they changed the beta coefficients of the exposure categories by more than 10% *Findings* Risk for combination feeding mothers to experience guilt was 6 times higher compared with exclusively breastfeeding mothers (RRR: .17, 95% CI: .10, .27)	Addressed a comprehensive range of potential confounders. Binary examination of guilt as being present or absent. Unrepresentative sample of women mainly from the UK and married or living with a partner. Participants asked about guilt via binary ‘yes/no’ response options to question, ‘have you ever felt guilty about the way you choose to feed your baby?’ (p .4). No definition of guilt provided
Lagan, B. M., Symon, A., Dalzell, J., & Whitford, H. (2014). ‘The midwives aren't allowed to tell you’: Perceived infant feeding policy restrictions in a formula feeding culture – The feeding your baby study. *Midwifery, 30*, 49–55. https://doi.org/10.1016/j.midw.2013.10.017	Explore the expectations and experiences of infant feeding	*Qualitative* Semi‐structured interviews and focus groups, cross‐sectional	Tayside, Scotland, UK, 38 participants took part in 7 focus groups and 40 interviews. 158 women approached, of which 78 women consented to participate	Ritchie and Lewis's (2003) five‐stage analytic framework approach, field notes were made to increase depth of data collected	*Descriptive statistics* Opportunity sampling was used to recruit participants. Participants were aged 19–41, 96% Caucasian, 92.3% married or in a relationship, 94.9% employed, 62.8% primiparous, *Qualitative themes* Mixed and missing messages; conflicting advice and information gaps; unrealistic preparation and breastfeeding pressure; emotional costs	Very limited exclusion criteria e.g. infant under care of social services or infant still in hospital at time of investigation. Understanding of questions was checked throughout interview process with participants to ensure study credibility and validity. Clear description of data analysis process. Inter‐rater reliability conducted by two independent researchers. Period between giving birth and taking part in the study varied between one to eight months across participants, potentially resulting in different emotional and/or practical infant feeding experiences. No definition of guilt provided
Lamontagne, C., Hamelin, A. M., & St‐Pierre, M. (2008). The breastfeeding experience of women with major difficulties who use the services of a breastfeeding clinic: A descriptive study. *International Breastfeeding Journal, 3*(17), 1–13. https://doi.org/10.1186/1746‐4358‐3‐17	Describe the breastfeeding experiences of women using breastfeeding services	*Mixed methods* *Quantitative* telephone questionnaire *Qualitative* semi‐structured interviews	Greater Quebec City, and Trois‐Riviéres, France *Quantitative* 86 participants *Qualitative* 12 participants Of 140 women approached, 54 did not participate (38.57% attrition)	*Quantitative* Frequency tables used to analyse questionnaire responses, descriptive statistics *Qualitative* Content analysis	*Descriptive statistics* Purposive sampling used to recruit participants to reflect diverse length of breastfeeding duration and of participant education level. Participants recruited through systematic sampling with a random start: 56% were aged 20–29, 98% were married or in common‐law, 55% had a university diploma, and 70% earned equal to or over $50,000 per annum *Quantitative* Participants recruited via systematic sampling with a random start using randomly selected records from clinic attendees, stratified by location. Most common reasons for breastfeeding cessation included: Latching difficulties/breast refusal (39%), and low milk supply (37%). Identified infant feeding support persons most commonly included: Partner (67%) and community center nurse (40%). Majority were satisfied with physicians (88%) and lactation consultants (94%) infant feeding support *Qualitative themes* Personal influences; social influences; breastfeeding clinic influence	Representative sample of participants with no significant differences regarding demographic characteristics. Small sample size recruited which did not allow for content saturation to be reached. Inclusion criteria clearly outlined in methodology; detailed outline of exclusion criteria considered. Clear rationale provided for inclusion of sociodemographic questions based on previously conducted research. Analysis steps well outlined in methodology, though there was no reported mention of inter‐rater reliability checking. Use of non‐validated research tools. No definition of guilt provided
Lee, E. (2007a). Living with risk in the age of ‘intensive motherhood’: Maternal identity and infant feeding. *Health, Risk and Society, 10*(5), 467–477. https://doi.org/10.1080/13698570802383432	Explore how mothers experience formula feeding in a context deeming formula milk as ‘risky’	*Mixed methods* *Qualitative* individual interviews, cross‐sectional *Quantitative* Telephone interviews conducted by NOP world, cross‐sectional	University of Kent, UK *Qualitative* 33 participants, no attrition reported *Quantitative* 504 participants, no attrition reported	*Qualitative* ‘… standard qualitative analysis techniques …’ (p. 472) *Quantitative* No information reported	*Descriptive statistics, qualitative* Opportunity sampling was used to recruit participants. Participants were aged 22–40, just under half were primiparous, half had infants 0–3 months old, half 4–6 months old *Descriptive statistics, quantitative* Quota sampling used to recruit participants, no descriptive statistics reported *Quantitative* Not breastfeeding was associated with guilt (33%), failure (32%), uncertainty about having done the right thing (48%), worries about what health visitor/midwife might say about formula feeding(23%), and worries about infant health consequences of formula feeding (20%) *Qualitative themes* Living with risk: Maternal identity and women who formula feed; moral collapse; confident mothers; defiant and defensive mothers; struggling with pressure and going it alone	Representative sample with no significant differences regarding demographic characteristics between participants. Part of a larger study conducted by the named author under the guidance of NOP world. Data splicing and missing information regarding data analyses. No definition of guilt provided
Lee, E. (2007b). Infant feeding in risk society. *Health, Risk and Society, 9*(3), 295–309. https://doi.org/10.1080/13698570701488910	Explore experiences with formula feeding	*Mixed methods* *Qualitative* individual interviews, cross‐sectional *Quantitative* Telephone interviews conducted by NOP world, cross‐sectional	London, South England, and the midlands *Qualitative* 33 participants, no attrition reported *Quantitative* 504 participants, no attrition reported	*Qualitative* ‘… standard qualitative analysis techniques …’ (p. 299) *Quantitative* No information reported	*Descriptive statistics, qualitative* Opportunity sampling was used to recruit participants. Participants were aged 22–40, just under half primiparous, half had infants 0–3 months old, half had infants 4–6 months old *Descriptive statistics, quantitative* Quota sampling used to recruit participants. 405 of 504 respondents had formula fed by the time of interview *Quantitative* Not breastfeeding was associated with guilt (33%), failure (32%), uncertainty about having done the right thing (48%), worries about what health visitor/midwife might say about formula feeding(23%), and worries about infant health consequences of formula feeding (20%) *Qualitative themes* Doing what is ‘easy’; worry, guilt, and failure; uncertainty	Representative sample with no significant differences regarding demographic characteristics. Part of a larger study conducted by the named author under the guidance of NOP world. Data splicing and missing information regarding data analyses. No definition of guilt provided
Lee, E. (2007c). Health, morality, and infant feeding: British mothers' experiences of formula milk use in the early weeks. *Sociology of Health and Illness, 29*(7), 1,075–1,090. https://doi.org/10.1111/j.1467‐9566.2007.01020.x	Aims to build upon insight about maternal experience with infant feeding in the early weeks following childbirth	*Mixed methods* *Qualitative* individual interviews, cross‐sectional *Quantitative* Telephone interviews conducted by NOP world, cross‐sectional	London, South England, and the midlands. *Qualitative* 33 participants, no attrition reported *Quantitative* 504 participants, no attrition reported	*Qualitative* thematic analysis *Quantitative* No information reported	*Descriptive statistics* Opportunity sampling used to recruit participants. Participants were aged 22–40, 14 primiparous, all used formula wholly or in part 0–3 months postpartum *Descriptive statistics, quantitative* 405 of 504 respondents had formula fed by the time of interview, half had infants 0–3 months old, half had infants 4–6 months old, 21% participants 16–24, 61% 25–34, 18% 35 or over *Quantitative* Quota sampling used to recruit participants. Not breastfeeding was associated with guilt (33%), failure (32%), uncertainty about having done the right thing (48%), worries about what health visitor/midwife might say about formula feeding(23%), and worries about infant health consequences of formula feeding (20%) *Qualitative themes* Feelings about feeding choices; failure, guilt, and worry; uncertainty and defiance; shame and avoidance; other mothers	Representative sample with no significant differences regarding demographic characteristics. Part of a larger study conducted by the named author under the guidance of NOP world. Data splicing and missing information regarding data analyses. No definition of guilt provided
Lee, E. & Furedi, F. (2005). Mothers' experience of, and attitudes to, using infant formula in the early months: Key findings. *School of Social Policy, Sociology and Social Research*, University of Kent, 1–8. Infant Formula‐main05.indd (researchgate.net)	Generate preliminary findings about how women experience feeding infant feeding in the current cultural and social context	*Mixed methods* *Qualitative* individual interviews, cross‐sectional *Quantitative* Telephone interviews conducted by NOP world, cross‐sectional	University of Kent, UK *Qualitative* 33 participants, no attrition reported *Quantitative* 504 participants, no attrition reported	*Qualitative* No information reported *Quantitative* No information reported	*Descriptive statistics, qualitative* Opportunity sampling used to recruit participants. All participants used formula wholly or in part 0–3 months postpartum *Descriptive statistics, quantitative* Quota sampling used to recruit participants. 405 of 504 respondents had formula fed by the time of interview *Quantitative* Not breastfeeding was associated with guilt (33%), failure (32%), uncertainty about having done the right thing (48%), worries about what health visitor/midwife might say about formula feeding(23%), and worries about infant health consequences of formula feeding (20%) *Qualitative themes* Attitudes towards formula use; pathways to formula use; feelings about formula use; interactions with healthcare professionals; interactions with family and other mothers	Representative sample with no significant differences regarding demographic characteristics. Part of a larger study conducted by the named author under the guidance of NOP world. Data splicing and missing information regarding data analyses. No definition of guilt provided
Mozingo, J., Davis, M., Droppleman, D. G., Merideth, A. (2000). ‘It wasn't working’: Women's experiences with short‐term breastfeeding. The American *Journal of Maternal and Child Nursing, 25*(3), 120–126. https://doi.org/10.1097/00005721‐200005000‐00004	Investigate the lived experiences of women who stop breastfeeding within the first 2 weeks postpartum	*Qualitative* Unstructured interviews, cross‐sectional	University of Tennessee, Knoxville, USA 9 participants, no attrition reported	Phenomenological approach, thematic analysis	*Descriptive statistics* Opportunity sampling used to recruit participants. Participants were aged 20–32, 8 were married, 7 primiparous, education ranged from high school to college graduate *Qualitative themes* Idealised expectations; clash with reality; personal feelings of discomfort; inadequate/inappropriate assistance; incremental disillusionment and breastfeeding cessation; relief versus guilt/shame/sense of failure; lingering self‐doubts versus resolution	No specified exclusion criteria regarding reasons for breastfeeding cessation within the first two weeks postpartum. Inter‐rater reliability checked by multiple members of the research team and analysis approach clearly outlined according to Pollio, Henley and Thompson (1997). Guilt and shame grouped in thematic analysis. No definitions of guilt or shame provided
Murphy, E. (2000). Risk, responsibility, and rhetoric in infant feeding. *Journal of Contemporary Ethnography, 29*(3), 291–325	Consider how mothers deal with threats to good mother identifies after breastfeeding cessation	*Qualitative* Semi‐structured interviews, longitudinal	Nottingham, England, 36 primiparous mothers, quota sample, no attrition reported	Inductive analysis with developed coding framework	*Descriptive statistics* Stratified sample of participants recruited based on occupational class and maternal age. Quota sampling was used to recruit participants. 35 White British, 11 ‘younger’ and 13 ‘older’ mothers recruited *Qualitative themes* The baby as unharmed by formula; beyond the mother's control; physical incapacity; blaming others	Information on occupational class profile of NHS practices were obtained to select participating practices. Large number (216) of interviews conducted longitudinally. Three interviews were not audio recorded but rather the researcher took field notes during interviewing which were written up immediately post‐interview. Method of analysis clearly outlined and inter‐rater reliability checked at weekly analysis meetings. No definition of guilt provided
Spencer, R. I., Greatrex‐White, S., & Fraser, D. M. (2014). ‘I thought it would keep them all quiet’. Women's experiences of breastfeeding as illusions of compliance: An interpretive phenomenological study. *Journal of Advanced Nursing, 71*(5), 1,076–1,086. https://doi.org/10.1111/jan.12592	Explore maternal experiences of breastfeeding	*Qualitative* Individual semi‐structured interviews, cross‐sectional	East midlands, UK 22 women, no attrition reported.	Interpretive phenomenological approach (Heidegger, 1962), Heideggerian interpretive phenomenology	*Descriptive statistics* purposive sampling approach used to recruit participants. Participants were aged 16–37, 59% primiparous, 21 women were White, British, 21 in a long‐term relationship, 1 had returned to work *Qualitative themes* Illusions of compliance; healthcare professional and society compliance; Family and friends compliance; Passive acquiescence; active decision‐making	Inclusion criteria for breastfeeding duration and infant age were well justified. Did not consider potential confounding variables as possible exclusion criteria. Method of data analysis clearly outlined. No evidence of inter‐rater reliability checking during analysis. No definition of guilt provided
Thomson, G., Ebisch‐Burton, K. & Flacking, R. (2015). Shame if you do – Shame if you do not: Women's experiences of infant feeding. *Maternal & Child Nutrition, 11*, 33–46. https://doi.org/10.1111/mcn.12148	Explore maternal experiences of infant feeding	*Qualitative* Focus groups and individual interviews, cross‐sectional	North West England, 63 women in 7 focus groups and 28 individual interviews, no attrition reported	Richie and Lewis's (2003) framework analysis	*Descriptive statistics* Opportunity sampling was used to recruit participants. Participants were aged 19–42, all bar one was married/cohabiting, majority were White, British *Qualitative themes* Vulnerability of subject (mother); Exposure of women's bodies and infant feeding; undermining and inadequate support; perceptions of inadequate mothering	No clear exclusion criteria for participant recruitment. No information reported regarding participant annual household income and education status. Participants recruited from areas of diverse deprivation. Interview schedule well outlined. Analysis checked by a second researcher. Niedenthal, Tangney, and Gavanski's (1994) Definitions of guilt and shame utilised. Lazare's (1987) categories of shame were used as a theoretical framework

### 3.1 Study quality

Missing statistical information (Chezem, Montgomery, & Fortman, 1997) and lack of interrater reliability testing (Crossley, 2009) may warrant caution regarding study credibility and transferability. Binary examination of guilt (Fallon, Komninou, Bennett, Halford, & Harrold, 2016; Komninou, Fallon, Halford, & Harrold, 2016) provides a reductionist view of this emotional experience, which lacks rich exploration of emotional experiences, and lack of survey item validity testing (Fallon, Komninou, et al., 2016) questions the content validity of examined constructs. Studies with unrepresentative samples (Chezem, Montgomery, & Fortman, 1997; Fallon, Komninou, et al., 2016; Komninou, Fallon, Halford, & Harrold, 2016) also limit generalisability of study findings. Lack of provided definitions of guilt and/or shame in included literature (Asiodu, Waters, Dailey, & Lyndon, 2017; Chezem, Montgomery, & Fortman, 1997; Crossley, 2009; Dalzell, 2007; Fahlquist, 2016; Fallon, Komninou, et al., 2016; Fox, McMullen, & Newburn, 2015; Hvatum & Glavin, 2017; Komninou, Fallon, Halford, & Harrold, 2016; Lagan, Symon, Dalzell, & Whitford, 2014; Lamontagne, Hamelin, & St‐Pierre, 2008; Lee, 2007a, 2007b, 2007c; Lee & Furedi, 2005; Mozingo, Davis, Droppleman, & Merideth, 2000; Murphy, 2000; Spencer, Greatrex‐White, & Fraser, 2014) and lack of conceptual use of terms are also problematic, as they potentially limit construct validity of terms and transferability of findings.

In the current review, four included articles engaged in data splicing and had missing information regarding data analyses (Lee, 2007a, 2007b, 2007c; Lee & Furedi, 2005). This methodological issue was overcome in the narrative synthesis by considering sample characteristics and results as a single unit during analysis. Studies not reporting clear exclusion criteria (Fox, McMullen, & Newburn, 2015; Thomson, Ebisch‐Burton, & Flacking, 2015) was problematic because motherhood involves diverse and complex experiences which may influence infant feeding outcomes, for example, traumatic birth (Garthus‐Niegel et al., 2017). By not utilising exclusion criteria, study findings were potentially vulnerable to sampling bias. Small study sample size (Lamontagne, Hamelin, & St‐Pierre, 2008) and some missing information regarding participant demographics (Crossley, 2009; Fahlquist, 2016; Thomson, Ebisch‐Burton, & Flacking, 2015) and study sampling strategy and study design (Hanell, 2017) may also limit transferability of findings.

### 3.2 Narrative synthesis of quantitative and mixed methods studies

Multivariate analyses were given reporting precedence over bivariate, univariate and descriptive analyses reported within included articles with quantitative components. Of the 8 included quantitative papers, only 2 quantitative studies used multivariate analyses.

### 3.3 Study descriptions and findings

#### 3.3.1 Examine the relationship between guilt and/or shame and different infant feeding outcomes

There were a total of 3 quantitative studies yielding 13 analyses (Chezem, Montgomery, & Fortman, 1997; Fallon, Komninou, et al., 2016; Komninou, Fallon, Halford, & Harrold, 2016) and 4 mixed methods studies yielding 1 analysis (Lee, 2007a, 2007b, 2007c; Lee & Furedi, 2005) which addressed research question (a).

#### 3.3.2 Breastfeeding

There were no significant differences between guilt scores of women with exclusive breastfeeding intentions and women with combination feeding intentions during pregnancy (Komninou, Fallon, Halford, & Harrold, 2016). However, postnatally, risk of guilt was 6 times higher for combination feeders compared with exclusive breast feeders (Adjusted RRR: 0.17, 95% CI: 0.10, 0.27).

#### 3.3.3 Formula feeding

Risk of guilt was 7 times lower for formula feeding women who had had exclusive formula feeding intentions during pregnancy (Adjusted RRR: 0.14, 95% CI: 0.08, 0.26) and 2 times lower for women with combination feeding intentions (RRR: 0.48, 95% CI: 0.29, 0.79), compared with women who had had exclusive breastfeeding intentions in pregnancy but whom were exclusively formula feeding postpartum (Fallon, Komninou, et al., 2016). Risk of guilt was 4 times lower for women who had exclusively formula fed since birth (Adjusted RRR: 0.45, 95% CI: 0.25, 0.79), and 2 times lower for combination feeders since birth (Adjusted RRR: 0.38, 95% CI: 0.21, 0.64) compared with women who initiated exclusive breastfeeding but whom were exclusively formula feeding postpartum (Fallon, Komninou, et al., 2016).

In bivariate analyses, not meeting breastfeeding intentions was associated with significantly higher guilt compared with women who met antenatal goals when returning to work within 1 year postpartum (*p* = .004; Chezem, Montgomery, & Fortman, 1997). In descriptive analyses, 33% of exclusively formula feeding women with antenatal breastfeeding intentions felt guilty in relation to their infant feeding method (Lee, 2007a, 2007b, 2007c; Lee & Furedi, 2005).

#### 3.3.4 Summary

Guilt was experienced more frequently by formula feeding mothers compared with combination feeding (Fallon, Komninou, et al., 2016) and breastfeeding (Komninou, Fallon, Halford, & Harrold, 2016) mothers. Guilt was also more pronounced when antenatal breastfeeding intentions were unmet (Chezem, Montgomery, & Fortman, 1997; Lee, 2007a, 2007b, 2007c; Lee & Furedi, 2005).

### 3.4 Framework synthesis of qualitative, mixed methods and quantitative studies

**FIGURE 2:**
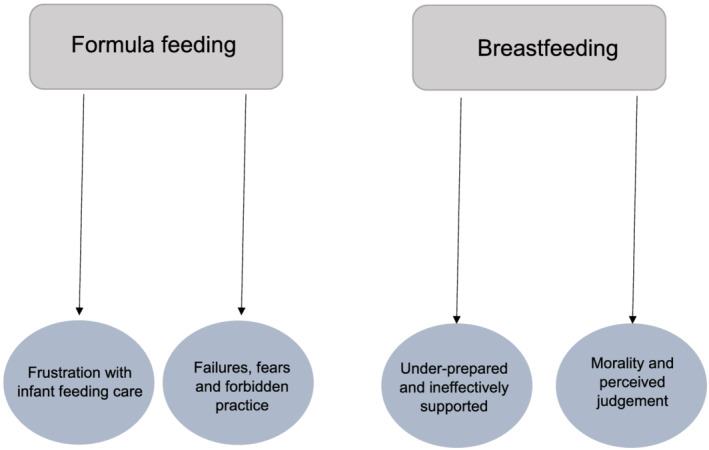
Diagrammatic overview of framework synthesis structure

Framework synthesis identified four themes split by infant feeding method (breastfeeding and formula feeding mothers), to answer research question (b). The search strategy identified 4 studies which were included in the breastfeeding analyses (Asiodu, Waters, Dailey, & Lyndon, 2017; Fox, McMullen, & Newburn, 2015; Hanell, 2017; Spencer, Greatrex‐White, & Fraser, 2014), 11 studies which were included in the formula feeding analyses (Crossley, 2009; Fahlquist, 2016; Hvatum & Glavin, 2017; Lagan, Symon, Dalzell, & Whitford, 2014; Lamontagne, Hamelin, & St‐Pierre, 2008; Lee, 2007a, 2007b, 2007c; Lee & Furedi, 2005; Mozingo, Davis, Droppleman, & Merideth, 2000; Murphy, 2000) and 2 studies which were included in both the breastfeeding and formula feeding analyses as they sampled across both feeding methods (Dalzell, 2007; Thomson, Ebisch‐Burton, & Flacking, 2015). Each theme is presented alongside illustrative quotes. Where verbatim quotes are used, these retained the nonidentifying label (e.g., pseudonym) used within the given study. Figure 2 provides a diagrammatic overview of the thematic structure.

### 3.5 Examine how guilt and/or shame are experienced differentially depending on infant feeding method

Framework synthesis identified 2 major themes from 6 studies for breastfeeding mothers: ‘underprepared and ineffectively supported’ and ‘morality and perceived judgement’. Due to only 2 included studies examining experiences of combination feeding mothers, findings from combination feeding and exclusively breastfeeding mothers were collapsed into the category *breastfeeding mothers*.

#### 3.5.1 Underprepared and ineffectively supported

Mothers perceived that health professionals ineffectively prepared them for postpartum breastfeeding challenges and postnatal experiences were consequentially often at odds with prenatal expectations (Fox, McMullen, & Newburn, 2015). This disparity led to feelings of self‐doubt, anxiety (Thomson, Ebisch‐Burton, & Flacking, 2015) and undermined breastfeeding self‐efficacy, ‘In the hospital they kept repeating that it shouldn't be painful, if you are doing it right it shouldn't hurt. And that wasn't particularly helpful, because it was painful for me’ (p. 6, Mother; Fox, McMullen, & Newburn, 2015).

Feeling unprepared for breastfeeding challenges also led to feelings of guilt (Asiodu, Waters, Dailey, & Lyndon, 2017) and shame (Hanell, 2017) when antenatal expectations were unreflective of postpartum experiences, ‘I broke down. It's like oh I can't make enough [breast milk] to feed my baby like that's what I'm supposed to do …’ (p. 870, postpartum participant; Asiodu, Waters, Dailey, & Lyndon, 2017), ‘I still want to breastfeed her until she is, at least breastfed exclusively until she's five or six months … Because [otherwise] it's one of those shame things.’ (p. 237, Veronica; Hanell, 2017).

Breastfeeding women also felt shame in response to perceptions of overinvolved care and nonconsensual breast handling by healthcare professionals (Thomson, Ebisch‐Burton, & Flacking, 2015). Breastfeeding mothers would have instead preferred to receive more hands‐off, practical support and also expressed a preference to have received more individualised infant feeding support (Dalzell, 2007). Breastfeeding mothers felt ineffectively supported by critical comments made by healthcare professionals about their infant and maternal shortcomings (e.g., their breasts or nipples being ‘too big’ or ‘too small’), which was associated with feelings of shame, ‘[Midwife] literally just got hold of it [breast], squeezed it and went like that [demonstrating the action] I was mortified …’ (p. 38, Lorraine; Thomson, Ebisch‐Burton, & Flacking, 2015), ‘Staff should observe feeding; being shown sooner may have helped’ (p. 81, M18; Dalzell, 2007).

#### 3.5.2 Mortality and perceived judgement

In most included qualitative studies, breastfeeding mothers felt morally obliged to adhere to ‘breast is best’ discourse, which was associated with guilt when breastfeeding difficulties were experienced (Fox, McMullen, & Newburn, 2015). Quantitative analysis also identified that guilt was equally likely to be experienced in association with internal as with external factors, with 37.6% of breastfeeding women experiencing internal (feelings of guilt originating from how one feels about their infant feeding method) and 32.7% of breastfeeding women experiencing external (feelings of guilt originating from how one perceives others to feel about their infant feeding method) guilt. Guilt was, however, also felt through both internal and external channels for 26.7% of mothers (Komninou, Fallon, Halford, & Harrold, 2016). It was identified commonly in the framework synthesis that trying and failing to breastfeed were more morally acceptable than formula feeding from birth, and alternative feeding methods were often perceived as wrongful (Spencer, Greatrex‐White, & Fraser, 2014), ‘I couldn't help but feel that I was sort of, I wasn't doing my job properly, if I didn't at least give it my absolute best shot’ (p. 6, Mother; Fox, McMullen, & Newburn, 2015).

Formula feeding was equated with inadequate mothering (Dalzell, 2007) and was commonly associated with loss of maternal identity (Hanell, 2017) in the framework synthesis. Some mothers felt the need to defend their infant feeding choice to maintain positive maternal identities, if exclusive breastfeeding were not possible, ‘I mean giving him one formula bottle like every couple of nights, is that still exclusively breastfeeding? … I don't like that because it makes me feel like, oh it's not enough. But I know it's enough because 99.99.99% of his meals are from my boob’ (p. 870, postpartum participant; Asiodu, Waters, Dailey, & Lyndon, 2017). ‘There is definitely elements of you're a better mother if you breast feed’ (p. 53, M6; Dalzell, 2007),

Perceived judgement influenced maternal feelings of self‐blame. Indeed, many women feigned effortless breastfeeding experiences, which were often at odds with their actual private experiences, in fear of being judged as a bad mother by healthcare professionals (Spencer, Greatrex‐White, & Fraser, 2014) or by family members (Hanell, 2017). Judgemental comments regarding breastfeeding from family and friends (Fox, McMullen, & Newburn, 2015) led to social sphere withdrawal (Thomson, Ebisch‐Burton, & Flacking, 2015). Quantitative analysis also provided evidence for the relationship between guilt and social support networks, with 58.7% of breastfeeding women experiencing external guilt in relation to family and 31.7% experiencing external guilt in relation to other mothers (Komninou, Fallon, Halford, & Harrold, 2016). The following illustrative quotes support this argument, ‘The other midwives, they were all nice, they was all oh how are you getting on and that and she's putting on weight, all fine all fine and I was thinking, it's not though, she's always not latching on properly… I didn't want to cry and [healthcare professionals] to think I wasn't coping’ (p. 1080, Jenny; Spencer, Greatrex‐White, & Fraser, 2014), ‘… I started to cry in front of my dad too. […] Because I do want to be able to breastfeed. And be a good mother … [dad's] not judging me, but I, it felt like that’ (p. 241, Veronica; Hanell, 2017).

Breastfeeding mothers resisted seeking help and often spoke of fearing being perceived as a failure. This was often discussed by women experiencing guilt in the context of breastfeeding pressure (Spencer, Greatrex‐White, & Fraser, 2014). Lack of public breastfeeding exposure resulted in shame and contradicted breastfeeding efforts (Thomson, Ebisch‐Burton, & Flacking, 2015). Several respondents spoke of avoiding help‐seeking behaviour due to perceived breastfeeding pressure, ‘I daren't say I've got problems because [other mothers] would go in to a whole “oh breast is best”…’ (p. 1080, Kelly; Spencer, Greatrex‐White, & Fraser, 2014). ‘I was more concerned with people looking and thinking … she should be [breastfeeding] somewhere behind closed doors …’ (p. 38, Ava; Thomson, Ebisch‐Burton, & Flacking, 2015).

### 3.6 Formula feeding mothers

Framework synthesis identified 2 major themes from 9 studies which examined the experiences of formula feeding women: ‘frustration with infant feeding care’ and ‘failures, fears and forbidden practice’.

#### 3.6.1 Frustration with infant feeding care

Inconsistent guidance and support (Lamontagne, Hamelin, & St‐Pierre, 2008) were perceived as frustrating and confusing (Lagan, Symon, Dalzell, & Whitford, 2014), and there was an expressed need for better quality in infant feeding care. Healthcare professionals were quick to blame mothers for breastfeeding difficulties, which led to feelings of guilt for women who were unable to breastfeed and who, subsequently, were formula feeding at the time of investigation (Fahlquist, 2016). Quantitative analysis also found that 64% of formula feeding women experienced external guilt in relation to healthcare professionals (Fallon, Komninou, et al., 2016). Feeling undermined by healthcare professionals and publicly embarrassed was also mentioned by mothers experiencing guilt, ‘I felt awful, my daughter was crying, she didn't eat enough, lost weight, I panicked all the time and didn't know what to do. The child health center told me the problem was mine, I did something wrong … no one helped me, and everyone was just nagging about how good it is to breastfeed’ (p. 235, Mother; Fahlquist, 2016).

Lack of respect from healthcare professionals regarding maternal wishes to supplement with formula exacerbated feelings of guilt and shame (Hvatum & Glavin, 2017) and resulted in resentment being held towards healthcare professionals (Murphy, 2000), ‘My baby didn't gain in weight but lost 750g, but even then I wasn't allowed to give substitute. I got the understanding that there had to be a complete crisis first. Almost like they had to legalize it. It makes you feel even more unsuccessful’ (p. 3149, Mother 8; Hvatum & Glavin, 2017).

Mothers often felt frustrated with healthcare professional support (Murphy, 2000). Mothers also disliked time constraints experienced during care (Mozingo, Davis, Droppleman, & Merideth, 2000). Frustration with quality of care resulted in concealment of infant feeding method and provoked feelings of guilt (Lee, 2007b), ‘I was lying a lot, especially with the health visitor because every week … “still breastfeeding?” It got to a stage when I was like, “yeah still, still doing a bit but giving [baby] the formula at night‐time.” Because it was just the same question and they make you feel guilty’ (p. 304, Mother; Lee, 2007b).

#### 3.6.2 Failures, fears, and forbidden practice

Women experiencing guilt often internalised feelings that they were letting their baby down and feared potential infant health consequences of formula supplementation (Fahlquist, 2016), whereas shame was attributed to the self and experienced for seemingly having failed in front of other mothers (Crossley, 2009). Formula feeding often led to dissociation from one's maternal identity (Murphy, 2000) and defensiveness over infant feeding method (Lee & Furedi, 2005). Failing to breastfeed was also associated with self‐blame (Mozingo, Davis, Droppleman, & Merideth, 2000) and postnatal depression (Thomson, Ebisch‐Burton, & Flacking, 2015). Quantitative analysis also found that for formula feeding mothers, guilt was experienced more commonly in relation to internal feelings (30%) than in relation to external factors (12%). Guilt was, however, also felt through both internal and external channels for 55% of formula feeding mothers (Fallon, Komninou, et al., 2016). The following participant accounts reflect these findings that formula feeding was linked with internalised perceptions of the self as having failed to achieve good mothering status, ‘It was all “Well, I breast fed for two years,” “Well I breastfed for a year” … I said to Clare afterwards, they'll never speak to me again because I'm not a real Mum, you know’ (p. 307, Mother; Murphy, 2000), ‘I ended up suffering from quite severe postnatal depression, I have always wondered … if I could have breastfed would it have happened’ (p.41, Jill; Thomson, Ebisch‐Burton, & Flacking, 2015).

Formula feeding mothers often avoided help‐seeking behaviour and frequently spoke of fearing judgement for their infant feeding method from healthcare professionals and social support networks (Crossley, 2009; Lee & Furedi, 2005). Quantitative findings also demonstrated that 68% of formula feeding mothers experienced external guilt associated with other mothers (Fallon, Komninou, et al., 2016). Prohibition of formula discussions also led mothers to feel that formula feeding was forbidden and that there was pressure to breastfeed (Crossley, 2009; Lee, 2007b), ‘The antenatal class I had attended was heavily biased towards breastfeeding. For instance, in the session on feeding, a flip chart was put up and we were asked to list the advantages and disadvantages of feeding babies in particular ways. The midwife only wrote down the advantages of breastfeeding and ignored anyone who mentioned bottle‐feeding advantages’ (p. 81, in text; Crossley, 2009), ‘When no one talks about formula, and the paediatric nurse says that she cannot “promote” formula, you feel like a criminal, like you are doing something illegal’ (p. 236, Mother; Fahlquist, 2016).

## 4 DISCUSSION

This mixed methods systematic review aimed to address 2 research questions, ‘examine the relationship between guilt and/or shame and different infant feeding outcomes’ and ‘examine how guilt and/or shame are experienced differentially depending on infant feeding method’. A framework synthesis of qualitative and quantitative data and a narrative synthesis of quantitative data were utilised to address the research questions. The framework synthesis identified 4 key themes: ‘underprepared and ineffectively supported’, ‘morality and perceived judgement’ (breastfeeding), ‘frustration with infant feeding care’ and ‘failures, fears and forbidden practice’ (formula feeding).

### 4.1 Research question (A): Examine the relationship between guilt and/or shame and different infant feeding outcomes

Guilt occurred more frequently among exclusively formula feeding mothers than combination feeders (Fallon, Komninou, et al., 2016) and exclusive breast feeders (Komninou, Fallon, Halford, & Harrold, 2016). All studies with quantitative components (Chezem, Montgomery, & Fortman, 1997; Fallon, Komninou, et al., 2016; Lamontagne, Hamelin, & St‐Pierre, 2008; Lee, 2007a, 2007b, 2007c; Lee & Furedi, 2005) found guilt was more pronounced in formula feeding women when breastfeeding intentions were unmet.

Previous reviews have found depression (Dennis & McQueen, 2009; Dias & Figueiredo, 2015) and anxiety (Fallon, Bennett, et al., 2016; Grigoriadis et al., 2018) to be related to formula supplementation and early breastfeeding cessation. The current review extends this work to other domains of negative effect known to be associated with poorer breastfeeding outcomes, namely, guilt and shame. From a biological standpoint, depression and anxiety (Stuebe, Grewen, & Meltzer‐Brody, 2013) are suggested to adversely affect hormones necessary for breastfeeding (Lonstein, 2007). Oestrogen plays an important role in the process of milk ejection during breastfeeding (Uvnäs‐Moberg & Eriksson, 1996) and is lowered in women with postnatal depression (Harris, 1996). Similarly, women who do not breastfeed demonstrate elevated cortisol levels, heart rate and lowered oxytocin in response to external stressors, compared with breastfeeding women (Cox et al., 2015). Given the link between shame and postnatal depression in the current review, biological theories underlying the relationship between negative maternal affect and poorer breastfeeding outcomes might extend to include the roles of guilt and shame.

### 4.2 Research question (B): Examine how guilt and/or shame are experienced differentially depending on infant feeding method

#### 4.2.1 Underprepared and ineffectively supported

Previous literature has found that antenatal breastfeeding preparation fails to adequately prepare mothers for common breastfeeding difficulties, which has a negative emotional impact when postnatal challenges are experienced (Hoddinott, Craig, Britten, & McInnes, 2012; Trickey & Newburn, 2014). The current review identified the theme ‘under‐prepared and ineffectively supported’, which extends this evidence, finding that unanticipated and unaddressed breastfeeding challenges were associated with guilt and shame. Depicting a more realistic portrayal of common breastfeeding difficulties and providing strategies to overcome challenges may enhance maternal breastfeeding confidence and extend postnatal breastfeeding duration (Brown, 2016; Hoddinott, Craig, Britten, & McInnes, 2012; Trickey & Newburn, 2014). Additionally, providing more balanced infant feeding guidance may allow mothers to make more informed decisions about their infant feeding status and help to minimise guilt and shame experiences (Appleton et al., 2018; Blixt, Johansson, Hildingsson, Papoutsi, & Rubertsson, 2019; Ericson & Palmér, 2018).

Guilt and shame were also experienced by breastfeeding mothers in relation to receiving overinvolved care and nonconsensual breast handling by healthcare professionals, which was reflective of midwives providing support as ‘technical experts’ (Swerts, Westhof, Bogaerts, & Lemiengre, 2016). In line with current review findings, participants in Swerts, Westhof, Bogaerts, and Lemiengre's (2016) study viewed ‘technical experts’ as paternalistic and preferred a ‘skilled companions’ approach to receiving infant feeding care. This links with a recent mixed‐methods systematic review examining women's experiences of Baby Friendly Initiative (BFI) compliant care in the UK, which found that health professional support was highly influential to women's experiences of care but that current delivery in the UK may foster negative emotional experiences, including guilt, particularly for those who formula feed (Fallon, Harrold, & Chisholm, 2019). Although midwives desire to be ‘skilled companions’, they often find it difficult to provide this support due to resource constraints and work environment barriers (Burns, Fenwick, Sheehan, & Schmied, 2013; Dykes, 2005; Mclelland et al., 2015).

#### 4.2.2 Morality and perceived judgement

This theme is supported by existing literature highlighting that mothers frequently experience social and societal pressures to breastfeed through synonymous associations with ‘good mothering’ (Hunt & Thomson, 2017).This can lead to feelings of guilt, failure, fears of being judged and inhibition of help seeking behaviour (Regan & Brown, 2019; Taylor & Wallace, 2017; Williams, Donaghue, & Kurz, 2012; Williams, Kurz, Summers, & Crabb, 2013). It is therefore important to move away from moral‐based language to minimise negative emotions for those experiencing breastfeeding difficulties or early breastfeeding cessation. No quantitative literature examined shame in relation to infant feeding outcomes. This is concerning, given both its associations with negative breastfeeding experiences in qualitative literature, and its associations with postnatal depression and help‐seeking avoidance (Dunford & Granger, 2017). Future research should therefore aim to quantify the relationship between maternal shame and infant feeding outcomes.

#### 4.2.3 Frustration with infant feeding care

Formula feeding mothers commonly experienced external guilt in relation to perceived ineffective healthcare professional support (Fallon, Komninou, et al., 2016). Review findings were also reflected in existing literature suggesting that unbalanced and inconsistent formula feeding guidance was linked with feelings of frustration, confusion, shame, guilt and abandonment (Almeida, Luz, & Ued, 2015; Cescutti‐Butler, Hemingway, & Hewitt‐Taylor, 2019; Harrison, Hepworth, & Brodribb, 2018; Lakshman, Ogilvie, & Ong, 2010). Formula feeding mothers also expressed a desire for more information about safe formula supplementation (Appleton et al., 2018; Blixt, Johansson, Hildingsson, Papoutsi, & Rubertsson, 2019; Ericson & Palmér, 2018). Although it is important to promote and support breastfeeding, it is also necessary to ensure that formula feeding mothers have adequate emotional and practical support to feed their baby safely and responsively.

#### 4.2.4 Failures, fears and forbidden practice

Formula feeding mothers who experienced guilt were more prone to feelings of failure which were discussed in the context of ‘breast is best’ discourse. This may be explained by self‐discrepancy theory, which proposes that maternal guilt and shame result from discrepancies between one's actual and ideal self (Liss, Schiffrin, & Rizzo, 2012). This suggests a need for a more flexible promotional message which dissipates an ‘all or nothing’ breastfeeding mentality and instead focuses on a more incremental ‘every feed counts’ approach to providing breastfeeding support (Braimoh & Davies, 2014; Brown, 2016; Símonardóttir & Gíslason, 2018).

### 4.3 Limitations

The quality of included studies limited the ability to form firm conclusions. The majority of included quantitative literature did not report statistical analyses in full (Chezem, Montgomery, & Fortman, 1997; Lee, 2007a, 2007b, 2007c; Lee & Furedi, 2005), and one study lacked scale validity testing (Fallon, Komninou, et al., 2016), collectively suggesting caution should be taken regarding validity of findings. Some quantitative papers involved binary examination of guilt (Fallon, Komninou, et al., 2016; Komninou, Fallon, Halford, & Harrold, 2016). Binary examination of concepts is problematic as it provides a reductionist view of how guilt and shame are experienced within an infant feeding context. Future research should therefore aim to explore contributing factors and outcomes of guilt and/or shame, to gain a clearer narrative for these negative affective states within an infant feeding context.

Only 2 of the 20 included papers defined shame (Hanell, 2017; Thomson, Ebisch‐Burton, & Flacking, 2015), and 1 paper defined guilt (Thomson, Ebisch‐Burton, & Flacking, 2015), and both mixed methods and qualitative literature grouped guilt and shame in thematic analysis (e.g., Fahlquist, 2016). This is problematic due to the overlap between term definitions (Niedenthal, Tangney, & Gavanski, 1994) and the differing outcomes of guilt and shame (e.g., Hvatum & Glavin, 2017), which may question construct validity of concepts. Future research should therefore aim to create infant feeding specific definitions of guilt and shame to improve research homogeneity.

Some qualitative literature included unrepresentative samples of mainly White, highly educated, partnered, primiparous women of high socioeconomic status (Asiodu, Waters, Dailey, & Lyndon, 2017; Fox, McMullen, & Newburn, 2015; Hvatum & Glavin, 2017; Lagan, Symon, Dalzell, & Whitford, 2014; Lamontagne, Hamelin, & St‐Pierre, 2008; Mozingo, Davis, Droppleman, & Merideth, 2000; Murphy, 2000; Spencer, Greatrex‐White, & Fraser, 2014; Thomson, Ebisch‐Burton, & Flacking, 2015), limiting transferability of findings. Several included qualitative literature hadsome missing demographic information (Crossley, 2009; Fahlquist, 2016; Thomson, Ebisch‐Burton, & Flacking, 2015). Given the role that demographic variables play in determining breastfeeding outcomes, for example, higher educational attainment, being multiparous and being partnered have been associated with increased chances of breastfeeding exclusively postpartum (Yngve & Sjösröm, 2001), not reporting this information hinders the ability to form firm conclusions.

## 5 CONCLUSION

A mixed‐methods systematic review synthesising the findings from 20 papers examined how guilt and/or shame were related to different infant feeding outcomes and examined how guilt and/or shame were experienced differentially depending on infant feeding method. Quantitative findings suggest guilt is experienced more frequently as breastfeeding exclusivity declines, especially when breastfeeding intentions are unmet. For breastfeeding mothers, guilt was experienced in relation to family and peers, whereas for formula feeding mothers, guilt was experienced in relation to healthcare professionals and peers. Lack of quantitative exploration of shame in relation to infant feeding outcomes prompted suggestions for future research. Qualitative findings identified a need for more realistic, nonjudgemental and mother‐centred support to minimise guilt and shame experiences for those who breastfeed. For formula feeding mothers, providing practical support about how to feed safely and providing emotional support to those who are unable to meet their breastfeeding intentions is critical for maternal wellbeing. A shift is also recommended from a ‘6 months exclusive breastfeeding’ to an ‘every feed counts’ approach to providing breastfeeding support.

## CONFLICTS OF INTEREST

The authors declare that they have no conflicts of interest.

## CONTRIBUTIONS

LJ was responsible for study conceptualisation, methodology, data analysis and initial manuscript draft. JH independently quality assessed included articles to examine interrater reliability with LJ. VF independently data extracted included articles to assess interrater reliability with LJ. VF reviewed identified themes based on feasibility. LJ, VF, LDP and JH reviewed, edited and approved the final manuscript as submitted.

## APPENDIX A FULL ELECTRONIC SEARCH STRATEGY FOR PsycINFO

### A.1 Details of search strategy conducted for study screening and inclusion in PsycINFO database

Keywords used to search for articles included: ‘shame*’; ‘guilt*’; ‘stigma*’; ‘moral*’; ‘breastfeed*’; ‘breast feed*’; ‘breast‐feed*’; ‘bottle feed*’; ‘bottle‐feed*’; ‘infant feed*’; ‘infant‐feed’; ‘formula feed*’; ‘formula‐feed’; ‘combi* feed’; and, ‘human lactat*’. Boolean operators were used to blend keywords, and truncation was used to identify variations of each included keyword. The search strategy implemented to identify eligible articles was as follows: [‘breastfeed*’ OR ‘breast feed*’ OR ‘breast‐feed*’ OR ‘bottle‐feed*’ OR ‘infant‐feed*’ OR ‘infant feed*’ OR ‘formula feed*’ OR ‘formula‐feed* OR ‘combi* feed’ OR ‘human lactat*’] AND [‘Guilt*’ OR ‘Shame*’ OR ‘Stigma*’ OR ‘Moral*’].
